# Effects of roasting and steeping on nutrients and physiochemical compounds in organically grown naked barley teas

**DOI:** 10.1016/j.fochx.2024.101385

**Published:** 2024-04-15

**Authors:** Mariona Martínez-Subirà, Brigid Meints, Elizabeth Tomasino, Patrick Hayes

**Affiliations:** a University of Lleida – AGROTECNIO-CERCA Center, Av. Rovira Roure 191, 25198 Lleida, Spain; b Department of Crop and Soil Science, Oregon State University, Corvallis, OR, USA; c Department of Food Science &Technology, Oregon State University, Corvallis, OR, USA

**Keywords:** Barley, Roasted, Tea, Nutrients, Dietary fiber, Phytochemical compounds, Antioxidant activity, Color

## Abstract

Barley tea, a popular beverage with cultural traditions in East Asia, has long been esteemed for its flavor, aroma, thirst-quenching properties and perceived health benefits attributed to bioactive compounds. This study investigated the nutritional, bioactive, and antioxidant aspects of three commercial naked barley varieties, focusing on the impact of roasting and subsequent steeping for tea. Roasting did not affect total dietary fiber or β-glucan content. The process reduced sugar content and led to the disappearance of free amino acids, contributing to high levels of acrylamide and color changes. Roasting diminished total phenolic compounds, particularly flavonoids, resulting in reduced antioxidant activity. Metabolite analysis identified compounds in roasted grains that could influence tea flavor and aroma. Roasted barley tea made from these varieties was not a source of dietary fiber or antioxidants, but also contained no acrylamide. Therefore, consumers seeking such benefits from barley are urged to consume whole grain foods.

## Highlights

• Roasting whole grains causes modest modifications in nutritional composition.
• Fiber and antioxidants are eliminated when roasted grains are steeped for tea.
• Roasted grain is dangerously high in acrylamide.
• Steeping roasted grain for tea leads to no detectable acrylamide.
• The flavor, aroma, and thirst-quenching properties of barley tea merit more research.

## 1 Introduction

Barley (*Hordeum vulgare* L.), a cereal grain in the Poaceae family, is one of the earliest cultivated grains and remains an important cereal crop today (Jacobsen et al., 2006). The main use of barley is as animal feed, but it is also used as an ingredient in health food formulations and to make alcoholic beverages (beer and certain types of whiskey) (Baik & Ullrich, 2008). There are also nonalcoholic barley-based beverages, including roasted barley tea (Newman & Newman, 2006).

Roasted barley tea (referred to as “barley tea” in the rest of this report) is a popular beverage in East Asia, including Korea, China, and Japan as a thirst quencher in the summer (Tatsu, Matsuo, Nakahara, Hofmann, & Steinhaus, 2020). It is traditionally prepared from roasted barley grain by hot water extraction and is a caffeine-free beverage that is served cold or hot. Historically, barley was roasted and brewed at home (Newman & Newman, 2006). Today, commercial products (pre-made teabags and/or pre-packaged drinks) are widely available in East Asia and in specialty markets in North America. Health-promoting properties of barley tea are often attributed to phytochemicals present in the raw grain (Patra et al., 2023) without taking into account the possible effects of roasting and steeping of the roasted grain. There are mixed reports in the literature on the levels of major bioactive compounds, such as β-glucan and phenolic compounds, present in barley tea (Kulkarni, Yokota, Suzuki, & Etoh, 2008; Oh, Kim, Park, & Lee, 2015; Seo et al., 2015; Wang et al., 2023). Unique properties of barley tea include its flavor, aroma, and color and the roasting process is a primary determinant of these attributes (Schlörmann et al., 2019). Roasting typically involves the exposure of dry grain to dry heat for short periods of time (160–250 °C for 5–15 min), although some products are based on additional pre-treatments, including soaking of the raw grain in water (Joung, Kim, Kim, Hurh, & Baek, 2018). The roasting process produces Maillard reaction compounds, which are said to be responsible for much of the reputed antioxidant capacity of roasted barley as well as the enhanced digestibility of products made from it, due to starch gelatinization and denaturation of proteins (Der Duh, Yen, Yen, & Chang, 2001; Etoh et al., 2004; Sharma & Gujral, 2011).

Barley grains may differ in important morphological characters that may affect teas made from them. These include hull adherence (covered vs. naked (Taketa et al., 2008)); inflorescence type (two-row vs. six-row (Komatsuda et al., 2007)), which refers to the number of fertile florets per rachis node; and starch type (Baik & Ullrich, 2008), which refers to the amylose: amylopectin ratio (normal, waxy, high amylose). Furthermore, kernel color may contribute to the properties of roasted barley and tea brewed from it. Purple and blue grains contain anthocyanins and black grains contain melanin, contributing to the antioxidant properties of the grain (Abdel-Aal, Young, & Rabalski, 2006). Most barley teas in East Asia are prepared using covered, six-row, white aleurone types (K. Sato, Research Institute for Bioresoruces, Kurashiki Japan, personal communication), although there are also some specialty products based on naked, two-row and/or colored barleys (Tatsu et al., 2020). In principle the hull is not likely to contribute significant nutritional value, as it composed primarily of insoluble dietary fiber. However, the hull fraction contains phenolics (Martínez-Subirà et al., 2021) and it may contribute unique flavor and aroma properties (Tatsu et al., 2020).

Humans have historically consumed naked barley as a food product, and there is a rich culinary history, especially in the Himalayas and North Africa (Newman & Newman, 2006). Today, naked barley is primarily used for direct food applications (steamed grain, miso, noodles, etc.) whereas covered barley is used for animal feed and as a source of fermentable material for beer and certain distilled beverages (Meints, Vallejos, & Hayes, 2021). In recent years there has been growing interest in using naked varieties for making malt-based beverages, such as beer and spirits, because these barleys can offer new flavor frontiers and higher process efficiencies (Meints et al., 2021).

There is limited literature describing nutrient and phytochemical profiles of roasted barley teas: most published work has focused on the processing of roasted grain (Der Duh et al., 2001; Joung et al., 2018; Montanuci, Jorge, & Jorge, 2016; Seo et al., 2015); health benefits, including increased blood fluidity, skin warmth and cutaneous blood flow (Ashigai et al., 2018; Oh et al., 2015; Seo et al., 2015); and/or volatile profiles (Joung et al., 2018; Tatsu et al., 2020). We hypothesized that the principal nutrients and phytochemicals present in raw barley grain are carried through to tea prepared by steeping roasted grain. We determined the proximate nutritional and analytical profiles of commercial barley grain grown in the Pacific Northwest of the USA and proceeded to assess the effects of (1) roasting and (2) steeping of roasted grain for tea in terms of nutritional profiles, compositional changes, acrylamide formation, and antioxidant activity.

## 2 Material and methods

### 2.1 Chemicals and reagents

HPLC grade acetonitrile, 2-propanol, n-hexane, methanol, formic and acetic acid were purchased from Scharlau (Scharlab SL, Spain). Reference standards for the phenolic compounds analysis: Ferulic acid, caffeic acid, coumaric acid, vanillic acid, syringic acid and apigenin-7-O-glucoside were obtained from Extrasynthese (Genay, France), while *p*-hydroxybenzoic acid, sinapic acid, catechin, and procyanidin B2 were purchased from Sigma Aldrich (Steinheim, Germany). 2,2′-azobis(2,4-dimethylvaleronytril) and 6-hydroxy-2- 5,7,8-tetramethylchroman-2-carboxylic acid used in ORAC assay were obtained from Sigma Aldrich (Steinheim, Germany) and 2,2-diphenyl-1-picrylhydrazyl reagent were purchased from Alfa Aesar (Ward Hill, MA, USA) to DPPH test. All other reagents used were of analytical grade from Scharlau (Scharlab SL, Spain). Deionized water was obtained from a Milli-Q water purification system (Millipore, Bedford, MA, USA).

### 2.2 Barley samples

Three naked barley genotypes were selected for this study (Table 1). All were grown commercially under certified organic conditions. Streaker, a three-component blend developed at Oregon State University (Meints et al., 2015) was grown at Pleasant Grove, California. Karma (PI60205), a land race from the Himalayan region was grown at Chiloquin, Oregon. Tibet37 - a landrace of either Himalayan or North African origin (unpublished genetic and phenotype data) - was grown at American Falls, Idaho. Streaker and Karma were sourced from Hummingbird Wholesale (Eugene, Oregon https://hummingbirdwholesale.com/). Tibet37 was sourced from Oregon Grain and Bean (Harrisburg, Oregon https://opencorporates.com/companies/us_or/149231490). Grain of the three barleys used for this research was harvested in 2021.

**Table 1:** Organically grown naked barleys used for characterization of chemical and nutritional properties of grain, roasted grain, and tea.

Variety	Row type	Seed coat color	Starch type
Streaker	6	White, brown, blue	Non-waxy
Karma	6	Purple	Non-waxy
Tibet37	2	Black	Non-waxy

### 2.3 Sample preparation

Grain of each variety was divided into two portions; one portion was milled into flour using a cyclone mill (CT193 Cyclotec, Foss, Eden Prairie, MN USA) and kept in the dark at -20 °C until used for analyses. The other portion was used for roasting and brewing the barley tea. One hundred and twenty grams of each of the three barleys was roasted at 188 °C for 16 min in a coffee roaster (model MR-101 DAINICHI, Niigata, Japan). The roasted grains were ground using a cyclone mill. Forty grams of ground roasted barley was placed in a paper tea bag (Fenshine Tea Filters, 4“ x 6”) and transferred to a glass jar containing one L of deionized water at 100 °C. The tea bag was steeped for 30 min, stirring every 10 min. The tea bag was then removed and the brewed tea was cooled to room temperature prior to storage in 50 mL PTFE tubes at -20 °C until analysis.

### 2.4 Chemical analyses

#### 2.4.1 Proximate analysis and acrylamide concentration

Proximate analysis of raw and roasted grain of each of the three varieties, acrylamide analysis of roasted grain and tea of each of the three varieties, and proximate analysis of one tea were performed by OMIC USA Inc. (https://omicusa.com/). OMIC USA is a National Accreditation Board (ANAB) accredited laboratory that complies with ISO/IEC 17025/2017 using AOAC International official methods of analysis. A complete description of each analysis procedure is provided in Supplemental Table 1. Due to cost considerations, the only analysis that was replicated was dietary fiber of Streaker tea. Briefly, ash (923.03), moisture (AOAC 925.10), fat profile (996.01) protein (992.23), sugars (977.20 (modified)), mineral profile (2011.14 (modified)) and dietary fiber (985.29 /991.43 (f)) were analyzed by AOAC (1990) method. Carbohydrate and calories were measured by calculation as follows: carbohydrate = (100 - % ash - % total fat - % moisture - % protein) and calories = (4× calculated carbs) + (9× analyzed fat) + (4× analyzed protein). Acrylamide was determined according to Mastovska and Lehotay (2006).

#### 2.4.2 β-glucan

β-glucan was determined using the mixed-linkage β-glucan assay (K-BGLU) kit from Megazyme (Wicklow, Ireland) adapting the procedures for each type of sample (flour, toasted, and liquid samples) as specified in the assay protocol. β-glucan analyses were carried out in duplicate and the results are presented as mean values.

#### 2.4.3 Free amino acid and metabolite profiling via nuclear magnetic resonance (NMR)

The extraction of free amino acids and metabolites in flour and roasted barley was performed using methods outlined by Lin, Wu, Tjeerdema, and Viant (2007) and Ramakrishnan, Ridge, Harnly, Mazzola, and Luthria (2017). The process involved adding a Chloroform-Methanol-Water solvent to the samples, conducting ultrasonic-assisted extraction and centrifugation, followed by filtration and drying. The extracted samples were then dissolved in 50 mM sodium phosphate pH 7.0, 0.456 mM DSS and 10% D2O. Tea samples were combined with 4.56 mM d6-DSS in 100% D2O for final concentrations of 0.456 mM d6-DSS and 10% D2O in the sample. Nuclear Magnetic Resonance (NMR) profiling was performed using a Bruker 800 MHz Avance IIIHD NMR spectrometer, and data processing involved calibrated parameters and Chenomx software. Metabolites were identified and quantified, and the results are presented as mean values from two analytical replications (for more details see Supplementary Table1).

#### 2.4.4 Phenolic profile

Phenolic compounds were extracted and assessed using ultra-performance liquid chromatographic tandem mass spectrometric analysis. Free and bound phenolic compounds of raw and roasted grains were extracted as described in Martínez et al. (2018) subjected to a micro-elution solid-phase extraction (μSPE) (Waters, Milford, MA, USA) and analyzed by liquid chromatography. Teas were first concentrated under vacuum and extracted twice using ethyl acetate. The extracts were then dried over sodium sulphate, concentrated under vacuum to dryness and the residue was redissolved in methanol for analyses by liquid chromatography (Atoui, Mansouri, Boskou, & Kefalas, 2005). Quantification was based on a 0.01–22 μg/g calibration curve of commercially available standards, and the results were expressed as micrograms per gram of dry sample. The limits of detection (LOD) ranged from 0.004 to 0.058 μg/g and the limits of quantification (LOQ) from 0.011 to 0.173 μg/g. Total phenolic compounds were calculated by adding all phenolic compounds.

#### 2.4.5 *Antioxidant capacity* in vitro

The antioxidant activity was determined using two chemical-based methods, which were described in detail in Supplementary Table 1: DPPH radical scavenging activity was determined using the method of Shimada, Fujikawa, Yahara, and Nakamura (1992). The Oxygen Radical Absorbance Capacity (ORAC) was measured as described Huang, Ou, Hampsch-Woodill, Flanagan, and Prior (2002). Analyses were carried out in duplicate and the results are presented as mean values.

### 2.5 Color quantification

The *L*^*⁎*^*, a*^*⁎*^ and *b*^*⁎*^ chromatic values were measured with a Minolta CR-410 colorimeter (Konica Minolta Sensing Americas, Inc., NJ, USA). *L*^∗^ value corresponds to the lightness (varying between 0 for white to 100 for black), *a*^∗^ represents color values from red (positive) to green (negative values) and b* expresses values from yellow (positive) to blue (negative values). Considering the coordinates L* a* b*, the color is expressed through C* (differences in chroma) and H° (differences in hue). These parameters were determined as by Bicho, Leitão, Ramalho, and Lidon (2012). Color quantification was carried out in triplicate and the results are presented as mean values.

### 2.6 Statistical analysis

Unless otherwise indicated, the data reported are averages of two or three observation. Analyses were performed using JMP®Pro16 (SAS institute Inc., Cary NC). Tukey-Kramer's HSD (α = 0.05) was used for multiple comparisons. Principal Component Analysis (PCA) was used to graphically represent the association between genotype, processing (roasting and steeping) and metabolite profiling using standardized data.

## 3 Results and discussion

Barley tea made from roasted grain has been a popular beverage in East Asia for thousands of years. It is perceived as a healthy beverage with beneficial properties attributed to bioactive compounds present in barley grains (Patra et al., 2023). However, there are few reports describing detailed and sequential profiles of nutrients and phytochemicals in raw and roasted grains and teas. We therefore determined the nutrient and bioactive compound contents in grain of three organically grown commercial barley varieties differing in seed coat color, and proceeded to evaluate the effects of roasting and the impact of steeping on the nutritional composition and phytochemical contents of roasted grains and tea.

Nutritional components of raw grains, roasted grains and teas are shown in Table 2. Overall, the nutritional composition values obtained for raw grains are within the ranges reported for naked barley: protein (10.8–16.6 g/100 g), lipids (2.7–3.9 g/100 g) ash (1.9–2.4 g/100 g) sugars (2–4.2 g/100 g) calcium (0.03–0.06 g/100 g) potassium (0.36–0.58 g/100 g) sodium (0.01–0.08 g/100 g), and iron (36–85 mg/kg) (Newman & Newman, 2008). Recognizing that statistical comparisons are not possible due to a lack of replication, there are notable differences between varieties in terms of total carbohydrates, total proteins, calcium, potassium, and sodium. Organic naked barley grain composition is known to be influenced by genotype, location, crop year, and crop year/location interactions (Bunting et al., 2022). Because we used commercial varieties that were grown in different locations and in different seasons (fall vs spring-planted), we cannot determine if these nutritional differences are due to genotypic, environmental, or interaction effects. Tibet37 had the highest values for all parameters except carbohydrate and sodium. However, these differences may be of limited overall nutritional impact. According to the percent daily value (%DV) determined by the FDA for each nutrient (FDA, 2020), consumption of 100 g of any of the three barley varieties would provide similar levels of carbohydrates (26% DV on average), calcium (3% DV on average), potassium (11% DV on average) and sodium (0.4% DV on average). Protein values were more variable: Tibet37 would provide 30% DV while Streaker would provide 19%.

**Table 2:** Proximate analyses of raw and roasted grains of three organically grown naked barley varieties and one barley tea.

	Raw			Roasted			Tea
	Streaker	Karma	Tibet37	Streaker	Karma	Tibet37	Streaker
Calories (Kcal/100 g)	364.79	362.32	366.25	404.14	406.29	405.93	4.69
Ash (g/100 g)	1.89	1.77	2.04	2.15	2.07	2.33	0.05
Moisture (g/100 g)	10.55	11.55	10.51	0.74	0.77	0.75	98.94
Total Fat (g/100 g)	2.91	3.12	3.29	3.14	3.53	3.65	0.13
Saturated Fat	0.82	0.87	0.91	0.89	0.99	1.07	0.117
Monounsaturated Fat	0.37	0.51	0.46	0.42	0.60	0.60	<0.01
Polyunsaturated Fat	1.72	1.74	1.78	1.84	1.94	1.95	<0.01
Trans Fat	<0.01	<0.01	<0.01	<0.01	<0.01	<0.01	<0.01
Total Carbohydrate (g/100 g)	75.13	70.57	69.39	82.98	78.67	72.68	0.69
Total Sugar (g/100 g)	1.50	1.50	1.90	<0.50	<0.50	<0.50	<0.50
Sucrose	1.50	1.50	1.90	<0.50	<0.50	<0.50	<0.50
Fructose	<0.50	<0.50	<0.50	<0.50	<0.50	<0.50	<0.50
Glucose	<0.50	<0.50	<0.50	<0.50	<0.50	<0.50	<0.50
Lactose	<0.50	<0.50	<0.50	<0.50	<0.50	<0.50	<0.50
Maltose	<0.50	<0.50	<0.50	<0.50	<0.50	<0.50	<0.50
Protein (g/100 g)	9.52	12.99	14.77	10.99	14.96	20.59	<0.20
Minerals (mg/100 g)							
Calcium	43.40	35.20	48.00	49.10	38.80	51.40	<0.50
Potassium	517.00	462.00	548.00	581.00	516.00	547.00	13.20
Sodium	12.00	12.00	7.40	13.60	12.80	15.70	<2.00
Iron	4.90	3.70	4.10	5.50	4.30	5.00	<0.50

Analyses reported by OMIC USA Inc. on un-replicated samples.

As expected, roasting reduced moisture in all three varieties from an average of 10.9% to 0.8%. This substantial decrease led to modest increases in most other values, except total sugar and sucrose, which were reduced to <0.5 g/100 g. Roasting is known to alter nutritional content due to Maillard and other heat driven reactions (Mizukami, Yoshida, Isagawa, Yamazaki, & Ono, 2014; Seal et al., 2008; Yeager et al., 2022). There were notable increases in total protein and sodium for Tibet37 (Table 2). Montanuci et al. (2016) also observed significant increases in protein content in barley roasted at 220 °C and 240 °C, owing to the decrease in moisture content; their roasted grain protein values of 13.9 and 14.3 g/100 g, respectively, were higher than Streaker but lower than Karma and Tibet37. These authors also reported reductions in the levels of total sugar and specific sugars. There are few studies reporting mineral content in roasted barley. Anand et al. (2019) reported iron values similar to those we reported (4.74 mg/100 g), but calcium values that were 70 mg greater. The effect of roasting on minerals has been reported in other crops; however, it is unclear what the effect of roasting is on specific minerals. Oboh, Ademiluyi, and Akindahunsi (2010) showed significant increases in calcium, sodium, magnesium and zinc content and decreases in iron and potassium content after roasting in maize varieties. Conversely, roasting did not significantly affect mineral dialyzability in roasted samples of quinoa (*Chenopodium quinoa*), kiwicha (*Amaranthus caudatus*), and kañiwa (*Chenopodium pallidicaule*) (Repo-carrasco-valencia et al., 2010).

Although complete analyses of soluble dietary fiber and acrylamide were performed for all three barley teas (Table 3), the proximate nutritional analysis was conducted for only the Streaker barley tea, due to cost considerations (Table 2). Nutrition labels are available for pre-made commercial roasted barley tea-based products, but as many of these contain other ingredients (e.g. corn, rice, malt flour, chicory, *Cassiae semen* extract) or additives (e.g. sugar, fructose, maltodextrin, sodium bicarbonate, ascorbic acid, synthetic favor), they are not appropriate for comparison. Streaker barley tea is a low-calorie, hydrating beverage. The carbohydrate content was 0.69 g/100 g, with <0.5 g/100 g of total sugars; no sugar was added during its preparation. The total fat content was 0.13 g/100 g (FDA, 2020). Among the minerals analyzed, potassium stood out for its high content (13.2 mg/100 g) compared to others. However, this barley tea would be considered a low potassium food since it provides only 0.28% DV per 100 g of tea (FDA, 2020). Even though the roasted grain of the three varieties showed high levels of proteins, carbohydrates, and dietary fiber, limited nutritional value was found in the tea, likely due to dilution and steep time (Table 2).

**Table 3:** Total dietary fiber, β-glucan, and acrylamide levels in raw grain, roasted grain and tea made from three organically grown naked barley varieties.

		Total Dietary Fiber (g/100 g)	β-glucan (g/100 g)	Acrylamide (μg /kg)
Raw	Streaker	12.77	5.13 d	ND
	Karma	15.88	6.92 a	ND
	Tibet37	12.92	6.02 b	ND
Roasted	Streaker	13.23	5.41 cd	780
	Karma	15.28	6.88 a	320
	Tibet37	15.66	5.83 bc	1200
Tea	Streaker	<0.1	0.018 e	ND
	Karma	<0.1	0.017 e	ND
	Tibet37	<0.1	0.014 e	ND

ND = Not Detected. Means followed by the same letter are not significantly different between genotypes, based on Tukey-Kramer's HSD, α = 0.05

### 3.1 Total dietary fiber, β-glucan, and acrylamide

β-glucan, arabinoxylan, cellulose, glucomannan and lignin are referred to as total dietary fiber in barley grains. Of all the components of total dietary fiber in barley, β-glucan is probably the most important in terms of human diet and health benefits (Newman & Newman, 2008). β-glucan has been associated with a reduction in glycemic index and cholesterol levels (Izydorczyk & Dexter, 2008). These are the basis of health claims by the US Food and Drug Administration and the European Food Safety Authority (EFSA, 2011; FDA (US Food and Drug Administration), 2008). As shown in Table 3, total dietary fiber and β-glucan levels in raw grain were highest in Karma and lowest in Streaker and the difference was statistically significant. Total fiber contents were similar to those of other previously reported naked barleys (12.6–15.6 g/100 g) (Newman & Newman, 2008), while grain β-glucan values are modest compared to values in the literature, where values as high as 8–11% are reported (Izydorczyk & Dexter, 2008). These high values are generally from waxy endosperm barleys (Baik & Ullrich, 2008) whereas the three barleys in this study are non-waxy types. Dietary fiber and β-glucan values remained relatively constant after roasting but were negligible in all three barley teas (Table 3). Previous studies also reported that the levels of soluble β-glucan were constant in roasted barley grains at temperatures below 200 °C (Montanuci et al., 2016) and the amount of total β-glucan was not affected by the roasting process in different barley cultivars (Sharma, Gujral, & Rosell, 2011). The β-glucan contents in barley teas measured in this study was similar to the concentrations recently reported by Wang et al. (2023) (0.007–0.015 g/100 g). We extended the analysis from roasted barley to barley tea and our findings directly contradict the popular press descriptions of barley tea as a source of dietary fiber (See Supplementary Table 2 for a partial listing of websites and product labels).

Acrylamide levels in food products are of concerns for human health as this compound has various toxicological properties (EFSA, 2015). Before roasting, the acrylamide concentration was below the LOD in all barley grain samples (Table 3). It is well known that acrylamide is predominantly formed due to a Maillard reaction between asparagine and reducing sugars (Mottram, Wedzicha, & Dodson, 2002) during processing at high temperatures (≥ 120 °C) with low humidity – conditions that occur during frying, roasting, and baking (EFSA, 2015). The acrylamide concentrations in the roasted barley grain ranged from 320 to 1200 μg/kg. Tibet37 - which had the highest content of asparagine, glucose, and sucrose in the raw grain (Table 2 and Supplementary Tables 3 and 4) - also had the highest level of acrylamide in the roasted grain (Table 3). In general, the acrylamide levels detected in the roasted grains, especially in Tibet37 (Table 3) were elevated compared to results obtained by Mizukami et al. (2014) for commercial roasted barley grains for barley tea (50–410 μg/kg). The acrylamide concentrations we found are similar to those in commercial ground roasted coffee (70–1080 μg/kg (EFSA, 2015)). The Benchmark levels of acrylamide in foods reported in EU regulation 2017/2158 does not include recommendations for acrylamide levels in roasted barley grains for tea, however it specifies 400 μg/kg for roasted coffee and 850 μg/kg for instant coffee or coffee substitutes exclusively from cereals (EU, 2017). Given that acrylamide is present in roasted barley grains, especially in Tibet37 and Streaker, and its consumption would increase exposure to this toxic compound, we do not recommend direct consumption of roasted barley grain. The acrylamide present in roasted barley grains is reported to be transferred into the tea during brewing (Mizukami, Yoshida, & Ono, 2016). However, in our research, the steeping process resulted in an incomplete extraction from the barley grain and much lower concentrations were measured in teas compared to the roasted grains (Table 3). Even though acrylamide is highly soluble in water, different factors in roasting processing parameters (temperature, heating time, moisture level in raw materials, and pH) and tea preparation (the water/roasted grain ratio, the blend composition and roasting degree) have been shown to influence the levels of acrylamide in the final beverage (Mizukami et al., 2016). Further research is required to determine if other roasting and/or tea preparation protocols lead to the non-detectable levels of acrylamide in barley tea that we observed.

### 3.2 Free amino acids and metabolite profiling

Total free amino acid content and profiles were significantly different among genotypes (Supplementary Table 3). Tibet37 had the highest total content (15.2 mM), with glutamine being most abundant. Asparagine was the major amino acid in Streaker and Karma, but the highest asparagine content was found in Tibet37. The amount of free amino acids in cereals depends largely on the species, cultivar, and growing conditions (Žilić, Aktag, Dejan, & Gokmen, 2021). No free amino acids were observed in either the roasted grains or the teas, likely due to their roles as precursors of different molecules in the Maillard reaction initiated by the roasting process. According to Seal et al. (2008), the concentration of amino acids is the most important and rate limiting factor of Maillard reaction product formation in cereal products. Not only does roasting cause the loss of amino acids, but also the high temperatures during this process can deaminate and decarboxylate the amino acids as reported by (Cai, Zhu, Ma, Thakur, & Zhang, 2021). Aung, Kim, Kim, Shin, and Kim (2023) and Kim, Han, Lim, and Cho (2021) used intermediate steps such as germination and steaming before roasting, and observed that even though free amino acids decreased after roasting, they remained in sufficient concentrations to be detected in the roasted grains.

**Fig. 1:**
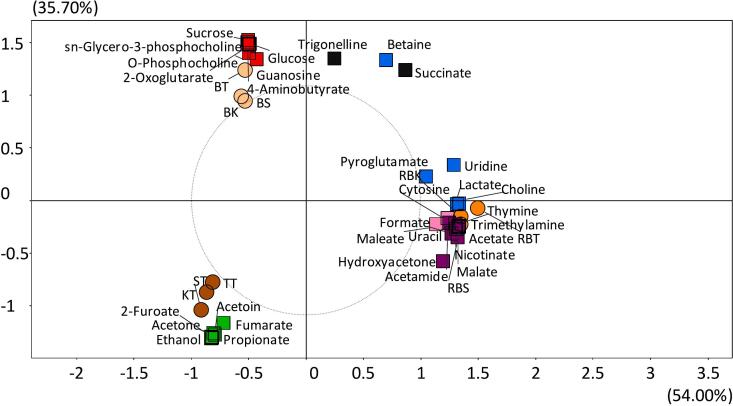
PCA showing metabolites detected in raw grain, roasted grain, and tea of three organically grown naked barley varieties. Light brown circles correspond to raw grain, orange circles to roasted grains, and dark brown circles to teas. The squares represent the metabolites. Metabolites in red were found only in raw grain, in pink those only in roasted grain, in green those only in tea, in blue those in flour, roast and tea, in violet those in roast and tea, and in black those in raw and roasted grain. BS: raw barley Streaker, BK: raw barley Karma, BT: raw barley Tibet37, RBS: roasted barley Streaker, RBK: roasted barley Karma, RBT: roasted barley Tibet37, ST: Streaker tea, KT: Karma Tea, TT: Tibet37 tea. (For interpretation of the references to color in this figure legend, the reader is referred to the web version of this article.)

As shown in Fig. 1 and Supplementary Table 4, some metabolites were detected in all three products (raw grains, roasted grains, and teas) while other metabolites were detected in only two-way combinations of products or in a single product. Betaine, choline, lactate, pyroglutamate (also known as pyroglutamic acid), and uridine were the only metabolites detected in all three barley products. Acetate, cytosine, formate, hydroxyacetone, malate, nicotinate, thymine, trimethylamine, and uracil were found in both roasted grains and teas, but not in raw barley. Succinate and trigonelline were found in raw and roasted grains, but not in teas. 2-Oxoglutarate, 4-aminobutyrate, guanosine, O-phosphocholine, sn-glycero-3-phosphocholine, and two free sugars - glucose and sucrose - were only detected in raw grains. Acetamide and maleate were found only in roasted grains, whereas other metabolites such as 2-furoate, acetoin, acetone, ethanol, fumarate, and propionate were only identified only in barley teas.

During roasting, sugars, amino acids, and other substances are converted into volatile and non-volatile chemicals through the Maillard reaction and caramelization processes and these may contribute to barley tea aroma and flavor. Several metabolites in barley tea were detected that may merit deeper exploration in terms of impacts on flavor and aroma. These compounds were also found to influence the aroma of coffees (Hu et al., 2020; Mortas, Gul, Yazici, & Dervisoğlu, 2017; Wei et al., 2012). 2-Furoate (detected only in the roasted teas) is the conjugate base of 2-furoic acid, which is abundant in roasted coffee (Mortas et al., 2017) and is responsible for the caramel-like notes (Wailzer, Klocker, Wolschann, & Buchbauer, 2016). Other metabolites - such as trigonelline, acetate and malate - play important roles in the differentiation terms of degree of roasting (Hu et al., 2020). We found that the trigonelline contained in raw grains was significantly reduced after roasting, corroborating prior reports (Baggenstoss, Poisson, Kaegi, Perren, & Escher, 2008). After heat treatment, trigonelline is largely broken down to nicotinic acid (Hu et al., 2020) and other volatile compounds such as pyridines and pyrroles, which are responsible for the nutty, roasted, walnut, toasty aromas (Seninde & Chambers, 2020). We observed a reduction in trigonelline after roasting and identified nicotinate (pyridinemonocarboxylate, the conjugate base of nicotinic acid) in the roasted grains and barley teas (Supplementary Table 4). Further analysis of volatile compounds is warranted, given that our results suggest that pyridines and pyrroles were possibly formed by trigonelline degradation and Maillard reactions, leading to burnt, roasted, and nutty aromas, as well as the bitter and astringent flavors of barley teas. These flavor descriptors were detected and reported in a separate paper on the sensory assessment of barley tea, and other non-alcoholic beverages, made from one of the three varieties (Streaker; Martínez-Subirà et al., unpublished data). Among the metabolites we detected in barley teas, the most abundant was acetate, with an average concentration of 0.12 mM. Malate was less abundant, at an average of 0.03 mM, compared to other metabolites. In coffee, these two organic acids have a low correlation with balance, flavor, aftertaste, and aroma (Hu et al., 2020). Other organic acids (lactate, formate and fumarate) and nucleic acid metabolites (uridine and uracil) were mainly identified in roasted grains and teas, and the relative metabolite levels of the samples were significantly different depending on the genotype. Betaine increased, and choline decreased, after the roasting process, in agreement with the findings of Park et al. (2019) in relation to roasted rice. Choline has been strongly and negatively correlated with sensory quality (Hu et al., 2020). Therefore, roasting would help decrease any potential off-flavors in the barley teas associated with this compound. Choline showed higher concentrations in roasted grains (1.97 mM on average) compared to raw barley grains (0.27 mM on average) and significantly lower concentrations in barley tea (0.04 mM on average) (Supplementary Table 4).

### 3.3 Phenolic compounds (PCs)

A total of 34 PCs were quantified in the raw grains using UHPLC-MS/MS (Table 4 and Supplementary Table 5). These included 20 phenolic acids and aldehydes, 8 flavan-3-ols and 6 flavone glycosides (Table 4). Phenolic compounds (PCs) of barley grain can be found free, conjugated with sugars via glycosidic bonds, or covalently linked to components of the cell wall (Li, Wang, Liu, Hong, & Zheng, 2023). Table 4 shows the PCs organized by main families in barley grain. The retention time, single reaction monitoring used for quantification, and fragment spectrum data of phenolic compounds identified in barley samples is shown in Supplementary Table 5.

**Table 4:** : Content of phenolic compounds and antioxidant capacity by DPPH and ORAC detected in raw grain, roasted grain and tea made from three organically grown naked barley varieties.

	Raw			Roasted			Tea			
	Streaker	Karma	Tibet37	Streaker	Karma	Tibet37	Streaker	Karma	Tibet37	*SED*
Phenolic compounds (μg/g)										
Catechin	59.51^b^	33.11^c^	66.05^a^							*0.52*
Catechin-glucoside	56.18^a^	32.18^b^	28.57^c^							*0.27*
Procyanidin B3	344.82^a^	179.23^b^	134.37^c^							*2.22*
Procyanidin B2	12.57^a^	2.79^c^	6.49^b^							*0.10*
GC-C/Prodelphinidin B4	187.81^a^	109.30^b^	84.68^c^							*1.20*
GC-C/Prodelphinidin B3	7.90^a^	5.50^b^	4.26^c^							*0.09*
Procyanidin-diglucoside	11.50^a^	6.15^b^	4.64^c^							*0.16*
Procyanidin C2	18.81^a^	8.39^b^	6.92^c^							*0.32*
**Total flavan-3-ols**	**699.10**^**a**^	**372.92**^**b**^	**333.66**^**c**^							***4.06***
Ap-6-C-ara-8-C-glu	0.07^c^	0.25^b^	0.65^a^							*0.14*
Isoscoparin-7-Glucoside	1.87^c^	2.51^b^	3.85^a^							*0.08*
Isoscoparin-7-rutinoside	0.32^c^	0.87^a^	0.48^b^							*0.04*
Isovitexin-7-Glucoside		0.42^a^	0.29^b^							*0.01*
Isovitexin-7-rutinoside	0.80^b^	0.92^a^	0.31^c^							*0.01*
**Total flavone glycosides**	**3.07**^**b**^	**5.16**^**a**^	**5.59**^**a**^							***0.18***
*p*-OHBenzoic acid	6.39^c^	11.97^b^	6.59^c^	17.99^a^	18.44^a^	11.83^b^	0.54^d^	0.44^d^	0.48^d^	*0.16*
OHBenzoic acid	0.91^a^			0.72^b^		0.61^c^	0.05^d^	0.05^d^	0.04^d^	*0.02*
2,4-DiOHBenzoic acid	0.09^e^	0.81^d^		1.63^c^	2.15^b^	2.52^a^	0.12^e^	0.18^e^	0.21^e^	*0.07*
*p*-Coumaric acid (*cis/trans*)	38.44^c^	60.05^a^	32.07	33.74^d^	47.99^b^	17.97^e^	0.05^g^	0.05^g^	4.63^f^	*0.82*
*m*-Coumaric acid (*cis/trans*)	6.41^a^	5.84^b^	3.22^c^	1.28^d^		0.29^e^	0.05^e^	0.04^e^	0.29^e^	*0.09*
Vanillic acid	3.50^e^	17.92^d^	22.88^c^	36.37^a^	31.51^b^	32.96^b^	0.49^f^	0.39^f^	0.51^f^	*0.44*
isoVA	11.98^a^			1.09^b^						*0.23*
Caffeic acid	0.27^d^	0.37^c^	0.35^c^	0.76^b^	0.72^b^	1.07^a^				*0.02*
Ferulic acid (*cis/trans*)	1195.38^a^	1043.74^b^	1215.88^a^	889.06^c^	751.94^e^	797.79^d^	1.28^f^	0.93^f^	0.92^f^	*11.41*
isoFerulic acid (*cis/trans*)	336.57^a^	236.01^b^	143.04^c^	35.30^d^	28.66^d^	29.38^d^	0.42^e^	0.29^e^	0.34^e^	*3.36*
8/5–5’-Diferulic acids	293.96^c^	459.27^b^	641.76^a^	80.18^e^	93.54^e^	176.47^d^			0.14^f^	*8.27*
TriFerulic acids	50.73^c^	88.32^a^	73.86^b^	19.77^e^	34.74^d^	34.66^d^				*1.50*
DiFA DC	51.22^c^	68.55^b^	84.44^a^							*1.50*
Syringic acid	2.24^f^	2.75^e^	6.84^c^	6.00^d^	7.40^b^	10.38^a^	0.16^g^	0.15^g^	0.38^g^	*0.14*
Sinapic acid	5.44^f^	18.63^d^	44.19^b^	15.75^e^	34.16^c^	61.91^a^				*0.71*
Cinnamic acid	0.25^d^	0.85^b^	0.53^c^	0.74^b^	1.38^a^	0.73^b^	0.03^e^	0.04^e^	0.04^e^	*0.04*
*p*-Coumaroyl-hexose	0.52^a^	0.35^b^	0.30^bc^	0.24^cd^		0.21^d^				*0.02*
Feruloyl-pentose	0.53^d^	1.25^c^	2.78^a^	1.99^b^	1.02^c^	2.03^a^				*0.07*
Caffeoyl-hexose	0.32^b^	0.13^d^	0.58^a^	0.14^d^	0.12^d^	0.20^c^				*0.01*
Sinapoyl-hexose	19.96^c^	25.15^b^	80.46^a^		4.13^e^	7.87^d^				*0.53*
Syringaldehyde	2.26^c^	4.80^b^	11.73^a^	0.94^e^	1.54^d^	4.77^b^	0.11^f^	0.14^f^	0.66^e^	*0.11*
**Total phenolic acids**	**2027.36**^**b**^	**2046.77**^**b**^	**2371.52**^**a**^	**1143.68**^**c**^	**1059.43**^**d**^	**1193.66**^**c**^	**3.30**^**e**^	**2.70**^**e**^	**8.65**^**e**^	***20.23***
**Total phenolic compounds**	**2729.53**^**a**^	**2424.85**^**b**^	**2710.77**^**a**^	**1143.68**^**c**^	**1059.43**^**d**^	**1193.66**^**c**^	**3.30**^**e**^	**2.70**^**e**^	**8.65**^**e**^	***22.44***

**Antioxidant activity**										
DPPH (%)	55.52^ab^	52.81^b^	57.43^a^	42.25^c^	37.72^d^	42.80^c^	1.88^e^	0.34^e^	2.31^e^	*1.11*
ORAC (μmol Trolox/g)	109.36^b^	96.90^c^	147.89^a^	82.78^d^	66.33^e^	112.85^b^	0.87^f^	0.77^f^	0.98^f^	*1.64*

Results are presented as mean of three replicates. Values within a row followed by different letters indicate significant differences according to Tukey-Kramer's HSD (0.05); SED, standard error of the difference.

Raw grains had the highest phenolic acids content, making up 74–87% of the total PCs as compared to flavan-3-ols and flavone glycosides (both flavonoids), which had percentages ranging from 13 to 26%. The most abundant PCs in flavan-3-ols were procyanidin B3 and prodelphinidin B4 (66–77% of the entire family), with the Streaker genotype having the highest contents of procyanidin B3 and prodelphinidin B4 (345 and 188 μg/g, respectively). The predominant phenolic acids were the ferulic acid monomer (*cis* and *trans*) and its dimers and trimers. The sum of all ferulic acids ranging between 1896 and 2159 μg/g of raw grain, with a higher content in Tibet37. *p*-Coumarics, sinapoyl-hexoside and sinapic acids were the next most abundant phenolic acid. Black barley (Tibet37) showed the highest phenolic acid content, while Streaker, with a percentage of white barley grain, had a high content of flavonoids. Similar results were also reported by Ge et al. (2021) who analyzed a different set of naked barleys.

The effect of roasting on the phenolic content of barley is shown in Table 4. Roasting caused a significant decrease in the total PC content of >55% in all genotypes. The reduction of PC in this study was similar to that reported after sand roasting (Sharma & Gujral, 2011). Twenty phenolic compounds were detected in the roasted grains; these included only phenolic acids. Neither flavan-3-ols nor flavone glycosides, both flavonoids, were detected. These results suggest that phenolic acids are comparatively more stable or resistant to roasting at 188 °C than flavonoids. The susceptibility to high temperatures varies with different classes of phenolic compounds due to differences in their chemical structures (Li et al., 2023). Previous studies have also found that flavonoids are more sensitive to heat and their degradation is observed at lower temperatures around 120 °C (Antony & Farid, 2022). Maghsoudlou, Asghari, and Tavasoli (2019) reported a significant decrease in catechin content after roasting related to its highly hydroxylated structure and sensitivity to redox reactions. The decrease of flavonoids in the three roasted barleys could be due to the oxidation and hydrolysis produced during roasting that would cause the decomposition of the flavonoids into simpler compounds, or even cause their degradation. Hydroxycinnamic and hydroxybenzoic phenolic acids were quantified in the roasted grains, but the total phenolic acids content decreased by around 47% during the roasting process. However, some minor compounds - such as *p*-OHbenzoic, 2,4-DiOHbenzoic, vanillic, caffeic, syringic, sinapic, and cinnamic acids - increased significantly after heat treatment. High temperature can cause dehydration and the appearance of chemical reactions in the roasted grains may be due to modification of the protein and lignocellulosic structures (Xu & Chang, 2008). This would favor the release of phenolic acids from the cellular matrix. However, it has been reported that the loss of phenolic acids predominates over their release from the matrix at temperatures above 120 °C for prolonged periods (≥20 min) (Li et al., 2023). The decrease of some phenolic acids in roasted grains can be attributed to the degradation, oxidation or polymerization of PCs during roasting. Syringaldehyde is derived from syringic acid and is susceptible to oxidation at high temperatures, resulting in a decrease in its content and an increase in syringic acid levels in three genotypes after roasting. Furthermore, the stability of phenolic acids also depends on their chemical structure. Those with a more stable structure, such as hydroxybenzoic acid derivatives, are generally more resistant to heating compared to hydroxycinnamic acid derivatives (Li et al., 2023), since the latter are more prone to thermal decarboxylation due to their carboxylic group attached to the side chain of acrylic acid (Bhinder et al., 2019). Our results corroborate these reports, since the phenolic acids lost after roasting were hydroxycinnamic acids (especially the main compounds such as ferulic and coumaric). The exceptions were, as noted previously, caffeic, sianpic and cinnamic acid which increased by 60%, 47% and 52%, respectively. The increase in caffeic acid after roasting could be due to the degradation of caffeoyl-hexose, a type of caffeoylquinic acid or chlorogenic acid which is thermally unstable and easily decomposes into quinic and caffeic acid (Maghsoudlou et al., 2019). Likewise, the increase in sinapic acid content could be explained by the degradation of the phenolic compound sinapoyl-hexose, resulting in the formation of sinapic acid and a sugar hexose. Cinnamic acid is the first molecule in the phenylpropanoid pathway and is the source of most hydroxycinnamic acid derivatives (Desjardins, 2008); the degradation of these PC could lead to an accumulation of cinnamic acid in roasted grains.

The total content of phenolics in barley teas ranged between 2.70 and 8.65 μg/g, with the highest content in Tibet37. Twelve phenolic acids were quantified in barley teas. These included benzoic, coumaric, and ferulic acids and their derivatives, vanillic, syringic, cinnamic acids and syringaldeyde. Five of these compounds have also been detected in highland barley teas, but information on concentration levels were not provided (Nie et al., 2022). Unlike Wang et al. (2023) who quantified total phenolics and total flavonoids in infusions based on roasted colored barley, we did not detect flavonoids in any of the teas we analyzed. Furthermore, we did not detect these compounds in roasted grains, a presumed consequence of their low stability during roasting. However, the total phenolic compounds content in our teas was similar to that reported by Wang et al. (2023). The content of phenolics in the roasted barley teas will ultimately be dependent on the barley grain matrix and which compounds can be extracted in a water-based solution, as many phenolics are more soluble in solvents rather than water (Cuevas-valenzuela, González-rojas, Wisniak, Apelblat, & Pérez-correa, 2014).

### 3.4 Antioxidant activity in vitro

Antioxidant activity was measured by DPPH radical scavenging activity and Oxygen Radical Absorbance Capacity (ORAC), two methods based on different mechanisms to provide the fullest picture of antioxidant activity of a food or beverage (Munteanu & Apetrei, 2021). The DPPH and ORAC values followed a trend similar to that observed for the PCs, indicating a positive correlation between the three assays (Table 4). Roasting caused a significant decrease in the antioxidant capacity of the three roasted barley samples. Both the DPPH and ORAC methods showed an average decrease of 26%. This could be explained by the reduction of PCs observed after roasting in the three genotypes, since these compounds are some of the main bioactive substances with antioxidant capacity in barley grains (Horvat et al., 2020). However, the antioxidant activity decrease was not as high as that observed for the PCs after roasting. This may be due the antioxidant reduction produced by loss of PCs being partially compensated for by the formation of compounds derived from pyrolysis, Maillard reaction and/or caramelization of carbohydrates during roasting (Turkmen, Sari, & Velioglu, 2005). These compounds and processes can strongly affect the antioxidant properties of the roasted material (Sharma & Gujral, 2011). Teas had much lower DPPH and ORAC values than roasted grains, which may be due to their relatively lower bioactive compound contents. No significant differences were observed in the antioxidant activity of the teas from the three varieties, although it was slightly lower in the Karma tea, following the same trend as observed for PCs content.

### 3.5 Color quantification

The analysis of chromatic parameters showed that lightness (L*) as well as parameters a*, b*, C* and H^0^ were significantly different between genotypes for raw grain, roasted grain, and tea (Table 5). The three genotypes differed in grain color, leading to significant differences between chromatic parameters. Raw Streaker was the only variety with a negative parameter a* value, corresponding to its overall greenish grain color, while raw Karma had the most positive a* value, being a purple variety. As was also expected, Tibet37 was the least luminous and yellowish (lower values of L* and b* respectively), since it has a black pigmentation in the pericarp.

**Table 5:** Mean values of colorimeter parameters for raw grains, roasted grains, and teas for three organically grown naked barley varieties.

		Colorimeter				
	Samples	L*	a*	b*	C*	*H*^*0*^
Raw	Streaker	85.48^a^	−0.28^i^	9.31g	9.31^g^	91.72^a^
	Karma	82.40^b^	1.58^g^	7.74^h^	7.90^h^	78.43^c^
	Tibet37	73.36^c^	0.51^h^	5.62^i^	5.64^i^	84.81^b^
Roasted	Streaker	52.43^d^	7.35^e^	17.92^d^	19.37^d^	67.69^f^
	Karma	50.40^e^	7.64^d^	16.80^e^	18.45^e^	65.56^h^
	Tibet37	46.80^f^	7.92^c^	15.64^f^	17.53^f^	63.15^i^
Tea	Streaker	43.98^g^	8.17^b^	23.69^a^	25.06^b^	70.97^e^
	Karma	46.93^f^	6.88^f^	23.37^b^	24.36^c^	73.59^d^
	Tibet37	40.37^h^	9.88^a^	23.20^c^	25.22^a^	66.92^g^

Different letters indicate significant differences after a mean comparison by Tukey-Kramer's HSD, α = 0.05

Lightness (L*) decreased significantly after roasting, whereas the parameters a* and b* increased significantly after processing, corroborating prior reports (Sharma et al., 2011; Yahya, Linforth, & Cook, 2014). The parameter C* (chroma) varied similarly to the chromatic coordinates a* and b*, with a large increase after roasting. The parameter H^0^ (hue), on the contrary, decreased sharply after roasting in all varieties. Based on the L*a*b* results, the same roasting protocol achieved different outcomes for each variety. Streaker was lighter, less reddish, and more yellowish than the other two varieties. Considering the C* and H^0^ values for each roasted grain, Streaker was also brighter and less brown than Karma and Tibet37. The compounds responsible for brown pigments of roasted grains were likely the water-soluble heterocyclic compounds and melanoidins that arose from the caramelization of sucrose and the Maillard reaction, respectively, during the roasting process (Yeager et al., 2022). Therefore, the initial color of the grain did not determine the color of the roasted grain. Rather, color differences were based on the composition and number of precursors for color-related reactions that occur during the roasting process. For example, Tibet37 had the highest content of free amino acids and reducing sugars and it was also the variety with the darkest roasted grain and darkest, reddest, and least yellowish tea. Although Streaker roasted grain was the lightest, its corresponding tea was darker and more reddish than Karma tea (an infusion with a less brown tone H^0^ = 73.59). In coffee, the color of the ground coffee does not always translate directly to the coloration of the coffee brew and it is well established that the brown color in roasted coffee is a result of melanoidins produced during the roasting process as well as the caramelization of sucrose (Yeager et al., 2022). Additional research on barley tea color may be warranted, due to the impacts of duration and temperature during roasting and steeping and the importance of color as an indicator of coffee quality and its role in sensory perception (Yeager et al., 2022).

## 4 Conclusions

This study delved into the nutritional, bioactive, and antioxidant aspects of three commercially available barley varieties, with a specific focus on the influence of roasting and brewing of tea from the roasted grain. Clearly, our results are limited to the barleys used and the subsequent roasting parameters (time, temperature, and moisture) and steeping process (time and dilution). Furthermore, the lack of replication of some analyses could limit their reliability and wider applicability. Focusing on our results, the roasting process - a crucial step in the production of barley tea - minimally altered nutritional composition but significantly reduced sugar contents. Although it did not affect total dietary fiber or β-glucan, it caused the disappearance of free amino acids, contributing to the formation of acrylamide and changes in color. The analysis of metabolites identified compounds present in roasted grains that could influence the final flavor and aroma of the tea. Roasting also reduced levels of total phenolic compounds, particularly flavonoids, resulting in decreased antioxidant activity. The steeping process is pivotal in shaping the ultimate composition and sensory characteristics of barley tea by extracting diverse compounds, including bioactive substances and flavor components, from the roasted barley grains. Barley tea is a low calorie, low carbohydrate beverage with no detectable levels of acrylamide. Based on proximate and chemical analyses, we reject the hypothesis that the beverages we prepared have bioactive properties. Despite popular press and advertising claims, barley tea, as prepared in this research, is not a source of dietary fiber or β-glucan. The steeping process extracted few phenolic compounds from roasted grains, leading to lower tea antioxidant activity. Consumers interested in increasing dietary fiber intake and benefiting from other bioactive properties of barley are encouraged to eat whole grain barley foods rather than drinking roasted barley tea-based beverages. Direct consumption of roasted barley grain is not recommended due to high levels of acrylamide. Barley tea color is not directly related to color of grain or roast and may be an important consideration as color can affect sensory perception. The continued popularity of barley tea is likely due to sensory perceptions including aroma, flavor, and its ability to quench thirst. This research underscores the complex factors affecting the nutritional quality and bioactive content of barley-based products, emphasizing the importance of understanding these transformations. This understanding is vital for optimizing the balance between flavor development and the retention of key bioactive compounds, particularly in barley teas. It offers consumers a nuanced perspective on how processing methods influence their dietary choices.

## Funding

This research was made possible by multiple donors to the Agricultural Research Foundation Fund for Barley Development. Mariona Martínez-Subirà was supported by a post-doctoral fellowship Margarita Salas funded by the European Union-Next Generation EU, Ministerio de Universidades y Plan de Recuperación, Transformación y Resiliencia, through the call of the 10.13039/501100009410University of Lleida (Spain) included in the I + D + i Project INNOBAR [PID2020-113009RB-I00], 10.13039/501100004837Spanish Ministry of Science and Innovation. Meints' time was supported by USDA-NIFA-OREI grant, [2020–51300-32179].

## CRediT authorship contribution statement

**Mariona Martínez-Subirà:** Writing – review & editing, Writing – original draft, Methodology, Formal analysis, Conceptualization. **Brigid Meints:** Writing – review & editing, Funding acquisition, Conceptualization. **Elizabeth Tomasino:** Writing – review & editing. **Patrick Hayes:** Writing – review & editing, Supervision, Resources, Methodology, Funding acquisition, Data curation, Conceptualization.

## Declaration of competing interest

The authors declare that they have no known competing financial interests or personal relationships that could have appeared to influence the work reported in this paper.

## Data Availability

Data will be made available on request.

## Appendix A Supplementary data

Effects of roasting and steeping on nutrients and physiochemical compounds in organically grown naked barley teas. Image 1
