# Supplementary material for: Effects of roasting and steeping on nutrients and physiochemical compounds in organically grown naked barley teas
Source: Food Chem X. 2024 Apr 15;22:101385. doi: 10.1016/j.fochx.2024.101385 (PMC11043873; doi:10.1016/j.fochx.2024.101385)
Supplement: Supplementary file 1 — Effects of roasting and steeping on nutrients and physiochemical compounds in organically grown naked barley teas. [file mmc1.docx]

## Supplementary data

**Effects of roasting and steeping on nutrients and physiochemical compounds in organically grown naked barley teas.**

Mariona Martínez-Subirà^a*^, Brigid Meints^b^, Elizabeth Tomasino^c^ and Patrick Hayes^b^

^a^ University of Lleida – AGROTECNIO-CERCA Center, Av. Rovira Roure 191, 25198, Lleida, Spain

^b^ Department of Crop and Soil Science, Oregon State University, Corvallis, Oregon USA

^c^ Department of Food Science &Technology, Oregon State University, Corvallis, Oregon USA

****Corresponding author at:*** University of Lleida - AGROTECNIO-CERCA Center, Av. Rovira Roure 191, 25198, Lleida, Spain. *E-mail addresses*: [mariona.martinez@udl.cat](mailto:mariona.martinez@udl.cat) (M. Martínez-Subirà)

| **Supplemental Table 1**: A complete description of proximate, acrylamide, free amino acids, metabolites profiling and antioxidant analysis procedure. | | |
| --- | --- | --- |
| Calories |  | Calories were measured by calculation following this equation: Calories = (4x calculated carbs) + (9x analyzed fat) + (4x analyzed protein) |
| Ash | AOAC 923.03* | Samples were incinerated in a muffle furnace (585°C) for at least 8 hours. |
| Moisture | AOAC 925.10* | Samples were dried in an oven (135°C) for 2 hours. |
| Fat Profile | AOAC 996.01* | Fat profile was analyzed according to AOAC 996.01 method. The samples were digested with heated HCl. Hydrolyzed fat components were subsequently extracted into ethyl ether and petroleum ether, which was then evaporated, saponified and methylated. Fatty acid methyl esters (FAME) were quantitatively determined by gas chromatography flame ionization (GC-FID). |
| Carbohydrates |  | Carbohydrate were measured by calculation following this equation: Carbohydrate = (100 - % ash - % total Fat - % moisture - % protein) |
| Sugar Profile | AOAC 977.20 (modified)* | Analysis of sugars was carried out according to AOAC 977.20 (modified). First, sugars were extracted from the sample with 1:1 water: methanol in a heated water bath (80-85°C for 30 minutes). The resulting sample extract was then centrifuged and filtered prior to analysis. Identification and quantification of sugar components (sucrose, fructose, glucose, lactose and maltose) was performed using a Liquid Chromatography Refractive Index detector (LC-RID), and summed for total sugar content. |
| Total Dietary Fiber | AOAC 985.29 /  AOAC 991.43 (f)* | Dietary fiber was calculated by subtracting the ash and protein from the initial dried residue, (AOAC 985.29 / AOAC 991.43 (f)). First, duplicate samples were gelatinized with heat-stable α-amylase in a heated water bath. The samples were then enzymatically digested with protease and amyloglucosidase to remove protein and starch. Ethanol was then added to precipitate soluble dietary fiber. The total residue was filtered and washed with ethanol and acetone. After drying in an oven, the residue was weighed. One duplicate sample was incinerated (500°C for 7 hours) while the other duplicate sample was analyzed for remaining protein (LECO). |
| Total Protein | AOAC 992.23* | Total protein was measured by combustion (AOAC 992.23 method) using a LECO Nitrogen Analyzer that utilizes a dual stage system operating at temperatures up to 1050°C to ensure complete combustion of organic samples. The samples were combusted in the presence of oxygen, and the gasses containing nitrogen oxides were further reduced to nitrogen (N_2_). The nitrogen was then measured via thermal conductivity detector and Protein results were then calculated based on the nitrogen response multiplied by a matrix specific protein factor (x 5.83 for barley). |
| Minerals Profile | AOAC 2011.14 (modified)* | Mineral profile was measured by AOAC 2011.14 (modified). The samples were pre-digested with concentrated nitric acid and hydrogen peroxide at ambient temperatures before digesting at 210°C in a closed vessel microwave accelerated reaction system (MARS). Sample solutions were infused into an Inductively Coupled Plasma Mass spectrometer (ICP-MS) to identify and quantify each element. |
| Acrylamide | Mastovska, et al., (2006) | Acrylamide was determined according to Mastovska et al., 2006. The samples were extracted with water and acetonitrile and defatted by shaking with hexane. After salt addition (magnesium sulfate and sodium chloride), acrylamide was partitioned into acetonitrile layer. A portion of the acetonitrile extract was cleaned using dispersive solid-phase extraction with magnesium sulfate and PSA (primary secondary amine). The detection and quantification were performed using isotopic analogue internal standard by Liquid Chromatography with Tandem Mass Spectrometer (LC-MS/MS). Limit of quantification of acrylamide concentration was 10μg /kg. |
| Free amino acids and metabolites profiling | Lin, et al., (2007) and Ramakrishnan, et al., (2017). | 2 mL of Chloroform-Methanol-Water (2:2:1.8) solvent was added to 0.2g of flour. The samples were subjected to ultrasonic-assisted extraction (300 W, 45°C, 60 s) and centrifuged (4000rpm, 10min). The supernatant was collected and the above process was repeated again with the sediment. The sample was filtered through a syringe containing a 0.45 µm polyvinylidene difluoride filter. Finally, the samples were kept for 9 h in a speed vac until dry. The extracted samples were dissolved in 0.6 ml of 50 mM sodium phosphate pH 7.0. 0.456 mM DSS. 10% D2O. Tea samples were combined with 4.56 mM d6-DSS in 100% D2O for final concentrations of 0.456 mM d6-DSS and 10% D2O in the sample.  NMR profiling was conducted using a Bruker 800 MHz Avance IIIHD NMR spectrometer equipped with a 5 mm TCI cryoprobe. Data were collected with a calibrated 90-degree pulse, a spectral window of 12.016 ppm, 76922 complex points, 512 scans, and a 1 s recycle delay. Experiments were collected in automation using a refrigerated SampleCase and IconNMR software (Bruker, Billerica, MA). Data were apodized, zero filled. Fourier transformed, and phased using the Chenomx software suite processor. Data were chemical shift referenced to 0.456 mM d6-DSS. Metabolites were identified and quantified using the Chenomx software suite profiler. Analyses were performed using two analytical replications and the results are presented as mean values. |
| DPPH radical scavenging activity | Shimada, et al., (1992) | 0.1 mL of extract or tea was added to 2.9 mL of DPPH in methanolic solution (0.09 mg/mL). The mixture was shaken by vortex mixer (Scientific Industries Inc., US) and stored for 30 min in the dark and measured at 517 nm spectrophotometrically (Shimadzu. Japan). The scavenging ability was calculated as: Scavenging activity (%) = [(ΔA517 of control − ΔA517 of sample)/ΔA517 of control] × 100. DPPH methanolic solution with methanol (barley extract) and with water (tea) is used as control. |
| ORAC | Huang, et al., (2002) | The determination of ORAC was carried out using a FLUOstar OPTIMA fluorescence reader (BMG Labtech) in a 96-well polystyrene microplate controlled by OPTIMA 2.10R2 software, working at 485 nm for excitation and 520 nm for emission. Trolox (6-hydroxy-2.- 5.7.8-tetramethylchroman-2-carboxylic acid) was used as control, with one ORAC unit being equal to the antioxidant protection given by 1 μmol Trolox. The antioxidant capacity of the extracts was calculated as micromoles of Trolox per gram of dry sample. |
| *AOAC International = ASSOCIATION OF OFFICIAL ANALYTICAL COLLABORATION. <https://www.aoac.org/> | | |

| **Supplementary Table 2**: A partial listing of web pages and advertisements of the benefits of barley tea. |
| --- |
| Barley Tea Health Benefits and Recipes – Why Barley Tea is Popular in Japan During Summer?. <https://thejapanstore.us/barley-tea/barley-tea-health-benefits/#SEC_05> Accessed 14.10.2023.  Barley Tea Benefits – 9 Reasons Why You Should Have This Drink. <https://healthwire.pk/healthcare/barley-tea-benefits/>Accessed 16.10.2023.  Barley Tea: Health Benefits, Side Effects And How To Prepare.<https://www.boldsky.com/health/wellness/health-benefits-of-barley-tea-134826.html> Accessed 20.10.2023.  Start your day with a cup of barley tea to get these 14 health benefits. <https://www.healthshots.com/healthy-eating/superfoods/what-makes-barley-tea-so-great-these-health-benefits-of-this-beverage-will-explain-why/> Accessed 23.10.2023. |

| **Supplementary Table 3:** Concentrations of free amino acids (mM) in raw grain of three organically grown naked barley varieties. | | | | | | | |
| --- | --- | --- | --- | --- | --- | --- | --- |
|  | **Streaker** | | **Karma** | | **Tibet37** | | **Average free amino acid content** |
| Alanine | 0.844 | b | 0.465 | c | 1.912 | a | 1.074 |
| Asparagine | 1.354 | b | 1.046 | c | 1.724 | a | 1.375 |
| Aspartate | 0.689 | a | 0.287 | c | 0.571 | b | 0.516 |
| Glutamate | 1.272 | b | 0.501 | c | 1.916 | a | 1.230 |
| Glutamine | 0.843 | b | 0.463 | c | 4.928 | a | 2.078 |
| Isoleucine | 0.105 | b | 0.084 | b | 0.350 | a | 0.180 |
| Leucine | 0.133 | b | 0.105 | b | 0.389 | a | 0.209 |
| Methionine | 0.066 | b | 0.027 | b | 0.199 | a | 0.097 |
| Phenylalanine | 0.123 | b | 0.056 | c | 0.204 | a | 0.128 |
| Proline | 0.628 | b | 0.715 | ab | 1.124 | a | 0.822 |
| Threonine | 0.224 | b | 0.157 | c | 0.649 | a | 0.343 |
| Tryptophan | 0.178 | a | 0.066 | c | 0.107 | b | 0.117 |
| Tyramine | 0.028 | a | 0.018 | b | 0.026 | ab | 0.024 |
| Tyrosine | 0.065 | b | 0.044 | b | 0.120 | a | 0.076 |
| Valine | 0.261 | b | 0.206 | c | 0.860 | a | 0.442 |
| β-Alanine | 0.050 | ab | 0.041 | b | 0.081 | a | 0.057 |
| Total | 6.862 | b | 4.281 | c | 15.159 | a | 8.767 |
| Means followed by the same letter are not significantly different between genotypes, based on Tukey-Kramer’s HSD. α = 0.05) | | | | | | | |

| **Supplementary Table 4:** Concentrations of metabolites (mM) in raw grains, roasted grains and teas of three organically-grown barley varieties. | | | | | | | | | | | | | | | | | | | | | | | | | | | | |
| --- | --- | --- | --- | --- | --- | --- | --- | --- | --- | --- | --- | --- | --- | --- | --- | --- | --- | --- | --- | --- | --- | --- | --- | --- | --- | --- | --- | --- |
|  | **Raw** | | | | | | | | | **Roasted** | | | | | | | | | **Tea** | | | | | | | | | |
|  | **Streaker** | | | **Karma** | | | **Tibet37** | | | **Streaker** | | | **Karma** | | | **Tibet37** | | | **Streaker** | | | **Karma** | | | | **Tibet37** | | |
| 2-Furoate |  |  |  |  |  |  |  |  |  |  |  |  |  |  |  |  |  |  | 0.004 | ± | 0.000 | 0.004 | ± | 0.001 | 0.005 | | ± | 0.000 |
| 2-Oxoglutarate | 0.174 | ± | 0.008 | 0.110 | ± | 0.006 | 0.228 | ± | 0.003 |  |  |  |  |  |  |  |  |  |  |  |  |  |  |  |  | |  |  |
| 4-Aminobutyrate | 0.292 | ± | 0.012 | 0.466 | ± | 0.021 | 0.332 | ± | 0.008 |  |  |  |  |  |  |  |  |  |  |  |  |  |  |  |  | |  |  |
| Acetamide |  |  |  |  |  |  |  |  |  | 0.095 | ± | 0.000 | 0.095 | ± | 0.000 | 0.203 | ± | 0.002 |  |  |  |  |  |  |  | |  |  |
| Acetate |  |  |  |  |  |  |  |  |  | 1.221 | ± | 0.108 | 1.404 | ± | 0.073 | 1.288 | ± | 0.107 | 0.133 | ± | 0.026 | 0.103 | ± | 0.005 | 0.110 | | ± | 0.005 |
| Acetoin |  |  |  |  |  |  |  |  |  |  |  |  |  |  |  |  |  |  | 0.001 | ± | 0.000 | 0.001 | ± | 0.000 | 0.001 | | ± | 0.000 |
| Acetone |  |  |  |  |  |  |  |  |  |  |  |  |  |  |  |  |  |  | 0.002 | ± | 0.000 | 0.004 | ± | 0.000 | 0.003 | | ± | 0.000 |
| Betaine | 2.568 | ± | 0.132 | 3.543 | ± | 0.035 | 2.661 | ± | 0.002 | 2.209 | ± | 0.000 | 3.348 | ± | 0.119 | 2.429 | ± | 0.027 | 0.077 | ± | 0.000 | 0.082 | ± | 0.001 | 0.059 | | ± | 0.002 |
| Choline | 0.132 | ± | 0.007 | 0.302 | ± | 0.006 | 0.366 | ± | 0.019 | 1.602 | ± | 0.000 | 2.159 | ± | 0.068 | 2.150 | ± | 0.022 | 0.047 | ± | 0.000 | 0.043 | ± | 0.001 | 0.042 | | ± | 0.002 |
| Cytosine |  |  |  |  |  |  |  |  |  | 0.089 | ± | 0.002 | 0.125 | ± | 0.007 | 0.209 | ± | 0.012 | 0.004 | ± | 0.000 | 0.004 | ± | 0.001 | 0.005 | | ± | 0.000 |
| Ethanol |  |  |  |  |  |  |  |  |  |  |  |  |  |  |  |  |  |  | 0.005 | ± | 0.000 | 0.004 | ± | 0.001 | 0.004 | | ± | 0.000 |
| Formate |  |  |  |  |  |  |  |  |  | 1.341 | ± | 0.040 | 0.967 | ± | 0.059 | 0.769 | ± | 0.002 | 0.097 | ± | 0.003 | 0.046 | ± | 0.000 | 0.038 | | ± | 0.002 |
| Fumarate |  |  |  |  |  |  |  |  |  |  |  |  |  |  |  |  |  |  | 0.005 | ± | 0.000 | 0.003 | ± | 0.000 | 0.001 | | ± | 0.000 |
| Glucose | 0.934 | ± | 0.056 | 0.793 | ± | 0.156 | 1.202 | ± | 0.120 |  |  |  |  |  |  |  |  |  |  |  |  |  |  |  |  | |  |  |
| Guanosine | 0.037 | ± | 0.001 | 0.010 | ± | 0.001 | 0.053 | ± | 0.000 |  |  |  |  |  |  |  |  |  |  |  |  |  |  |  |  | |  |  |
| Hydroxyacetone |  |  |  |  |  |  |  |  |  | 0.107 | ± | 0.000 | 0.093 | ± | 0.021 | 0.064 | ± | 0.005 | 0.035 | ± | 0.000 | 0.014 | ± | 0.001 | 0.011 | | ± | 0.001 |
| Lactate | 0.021 | ± | 0.006 | 0.034 | ± | 0.016 | 0.018 | ± | 0.000 | 0.151 | ± | 0.000 | 0.147 | ± | 0.005 | 0.107 | ± | 0.000 | 0.008 | ± | 0.000 | 0.006 | ± | 0.001 | 0.006 | | ± | 0.000 |
| Malate |  |  |  |  |  |  |  |  |  | 3.119 | ± | 0.073 | 2.683 | ± | 0.265 | 2.396 | ± | 0.016 | 0.040 | ± | 0.000 | 0.034 | ± | 0.003 | 0.023 | | ± | 0.002 |
| Maleate |  |  |  |  |  |  |  |  |  | 0.042 | ± | 0.000 | 0.031 | ± | 0.001 | 0.010 | ± | 0.001 |  |  |  |  |  |  |  | |  |  |
| Nicotinate |  |  |  |  |  |  |  |  |  | 0.060 | ± | 0.000 | 0.057 | ± | 0.005 | 0.042 | ± | 0.002 | 0.002 | ± | 0.000 | 0.001 | ± | 0.000 | 0.001 | | ± | 0.000 |
| O-Phosphocholine | 0.044 | ± | 0.003 | 0.043 | ± | 0.005 | 0.022 | ± | 0.003 |  |  |  |  |  |  |  |  |  |  |  |  |  |  |  |  | |  |  |
| Propionate |  |  |  |  |  |  |  |  |  |  |  |  |  |  |  |  |  |  | 0.005 | ± | 0.000 | 0.003 | ± | 0.000 | 0.004 | | ± | 0.000 |
| Pyroglutamate | 0.447 | ± | 0.014 | 0.229 | ± | 0.029 | 1.806 | ± | 0.130 | 1.897 | ± | 0.000 | 1.568 | ± | 0.057 | 5.277 | ± | 0.206 | 0.078 | ± | 0.000 | 0.044 | ± | 0.009 | 0.153 | | ± | 0.012 |
| sn-Glycero-3-phosphocholine | 0.103 | ± | 0.008 | 0.205 | ± | 0.004 | 0.217 | ± | 0.004 |  |  |  |  |  |  |  |  |  |  |  |  |  |  |  |  | |  |  |
| Succinate | 0.147 | ± | 0.011 | 0.107 | ± | 0.002 | 0.114 | ± | 0.001 | 0.129 | ± | 0.000 | 0.129 | ± | 0.000 | 0.131 | ± | 0.012 |  |  |  |  |  |  |  | |  |  |
| Sucrose | 11.409 | ± | 0.656 | 10.130 | ± | 0.490 | 15.908 | ± | 0.217 |  |  |  |  |  |  |  |  |  |  |  |  |  |  |  |  | |  |  |
| Thymine |  |  |  |  |  |  |  |  |  | 0.095 | ± | 0.007 | 0.090 | ± | 0.000 | 0.125 | ± | 0.010 | 0.004 | ± | 0.000 | 0.003 | ± | 0.000 | 0.004 | | ± | 0.000 |
| Trigonelline | 0.015 | ± | 0.001 | 0.016 | ± | 0.000 | 0.041 | ± | 0.001 | 0.008 | ± | 0.000 | 0.009 | ± | 0.000 | 0.024 | ± | 0.001 |  |  |  |  |  |  |  | |  |  |
| Trimethylamine |  |  |  |  |  |  |  |  |  | 0.162 | ± | 0.000 | 0.186 | ± | 0.014 | 0.170 | ± | 0.001 | 0.004 | ± | 0.000 | 0.004 | ± | 0.000 | 0.003 | | ± | 0.000 |
| Uracil |  |  |  |  |  |  |  |  |  | 0.067 | ± | 0.004 | 0.076 | ± | 0.001 | 0.113 | ± | 0.006 | 0.003 | ± | 0.000 | 0.002 | ± | 0.000 | 0.002 | | ± | 0.000 |
| Uridine | 0.082 | ± | 0.008 | 0.032 | ± | 0.001 | 0.083 | ± | 0.008 | 0.167 | ± | 0.006 | 0.166 | ± | 0.011 | 0.234 | ± | 0.014 | 0.006 | ± | 0.000 | 0.004 | ± | 0.001 | 0.005 | | ± | 0.000 |
| Total | 16.368 | ± | 1.001 | 16.510 | ± | 0.441 | 22.824 | ± | 0.429 | 12.560 | ± | 0.056 | 13.331 | ± | 0.577 | 15.739 | ± | 0.017 | 0.561 | ± | 0.029 | 0.409 | ± | 0.025 | 0.478 | | ± | 0.019 |
| Data showed as mean ± standard deviation | | | | | | | | | | | | | | | | | | | | | | | | | | | | |

| **Supplementary Table 5.** Retention time, MS and MS2 data of phenolic compounds identified in barley samples | | | | | | | | | | |
| --- | --- | --- | --- | --- | --- | --- | --- | --- | --- | --- |
|  | Rt (min) | [M-H]^-/+^  (*m/z*) | SRM transition^a^ | Cone Voltage (V) | Collision Energy (eV) | MS2 fragment spectrum^b^ | Quantified as | Identification level ^c^ | LOD  (µg/g) | LOQ  (µg/g) |
| **Flavan-3-ols** |  |  |  |  |  |  |  |  |  |  |
| Procyanidin diglucoside | 1.97 | 613 | 451 | 45 | 15 | 451/289 | Catechin | Tent |  |  |
| Prodelphinidin B4 (GC-C) | 2.78 | 593 | 289 | 45 | 20 | 289 | Procyanidin B2 | Tent |  |  |
| Prodelphinidin B3 (GC-C) | 4.21 | 593 | 289 | 45 | 20 | 467 / 289 | Procyanidin B2 | Tent |  |  |
| Procyanidin B3 (C-C) | 4.4 | 577 | 289 | 45 | 20 | 425/289 | Procyanidin B2 | Tent |  |  |
| Catechin-glucoside | 4.6 | 451 | 289 | 45 | 15 | 289 | Catechin | Tent |  |  |
| Procyanidin C2 (C-C-C) | 4.8 | 865 | 289 | 60 | 30 | 577/289 | Catechin | Tent |  |  |
| Catechin | 5.01 | 289 | 245 | 45 | 15 | 245/205/151 | Catechin | Std | 0.058 | 0.173 |
| Procyanidin B2 | 6.31 | 577 | 289 | 45 | 20 | 289 | Procyanidin B2 | Std | 0.033 | 0.100 |
| **Flavone glycosides** |  |  |  |  |  |  |  |  |  |  |
| Isoorientin (luteolin-6-C-glucoside) | 10.95 | 447 | 357 | 50 | 20 | - | Luteolin-7-glucoside | Tent |  |  |
| Isovitexin-7-O-rutinoside | 11.2 | 739 | 431 | 60 | 20 | 431/311 | Apigenin-7-O-Glucoside | Tent |  |  |
| Isovitexin-7-O-(6-sinapoyl)-glucoside-4'-O-glucoside | 11.34 | 961 | 799 | 60 | 20 | 799/593 | Apigenin-7-O-Glucoside | Tent |  |  |
| Apigenin-6-C-arabinoside-8-C-glucoside | 11.35 | 563 | 353 | 60 | 20 | 473/443/383/353 | Apigenin-7-O-Glucoside | Tent |  |  |
| Isovitexin-7-O-(6-feruloyl)-glucoside-4'-O-glucoside | 11.74 | 931 | 769 | 60 | 20 | - | Apigenin-7-O-Glucoside | Tent |  |  |
| Isoscoparin-7-O-rutinoside | 12.28 | 769 | 461 | 60 | 20 | 461/341 | Luteolin-7-glucoside | Tent |  |  |
| Apigenin-7-O-Glucoside | 16.4 | 431 | 269 | 50 | 25 | - | Apigenin-7-O-Glucoside | Std | 0.050 | 0.017 |
| **Phenolic Acids and Aldehydes** |  |  |  |  |  |  |  |  |  |  |
| 2.4-Dihydroxybenzoic acid | 2.77 | 153 | 109 | 45 | 15 | - | *p*-hydroxybenzoic acid | Tent |  |  |
| Caffeoyl-hexose | 3.25 | 341 | 179 | 40 | 15 | 179/135 | Caffeic acid | Tent |  |  |
| *p*-Coumaroyl-hexose | 3.24 | 325 | 163 | 35 | 10 | 119/93 | *p*-Coumaric acid *(trans*) | Tent |  |  |
| *p*-Hydroxybenzoic acid | 4.36 | 137 | 93 | 30 | 15 | - | *p*-hydroxybenzoic acid | Std | 0.005 | 0.014 |
| Sinapoyl-hexose | 5.3 | 385 | 223 | 60 | 25 | 223/205 | Sinapic acid | Tent |  |  |
| Vanillic acid | 5.59 | 167 | 123 | 30 | 10 | 152/123 | Vanillic acid | Std | 0.042 | 0.127 |
| Caffeic acid | 5.84 | 179 | 135 | 20 | 12 | 135 | Caffeic acid | Std | 0.036 | 0.107 |
| Iso- vanillic acid | 6.00 | 167 | 123 | 30 | 10 | 152/123 | Vanillic acid | Tent |  |  |
| Syringic acid | 6.33 | 197 | 182 | 30 | 10 | 182/153 | Syringic acid | Std | 0.004 | 0.011 |
| Feruloyl-pentose | 7.57 | 325 | 193 | 40 | 10 | 193/149/134 | Ferulic acid (*trans*) | Tent |  |  |
| *p*-Coumaric acid (*trans*) | 8.8 | 163 | 119 | 35 | 10 | 119/93 | *p*-Coumaric acid (*trans*) | Std | 0.007 | 0.021 |
| Syringaldehyde | 9.39 | 181 | 166 | 30 | 15 | 166/151 | *p*-Coumaric acid *(trans*) | Tent |  |  |
| Coumaric acid (*m*-CoA or *p*-CoA cis) | 10.28 | 163 | 119 | 35 | 10 | 119/93 | *p*-Coumaric acid *(trans*) | Tent |  |  |
| Ferulic acid (*trans*) | 10.99 | 193 | 134 | 30 | 15 | 178/134/149 | Ferulic acid (*trans*) | Std | 0.006 | 0.019 |
| Sinapic acid | 11.68 | 223 | 164 | 35 | 15 | 208/164/149 | Sinapic acid | Std | 0.054 | 0.163 |
| Hydroxybenzoic acid | 12.41 | 137 | 93 | 30 | 15 | 93 | *p*-hydroxybenzoic acid | Tent |  |  |
| Iso-Ferulic acid | 12.64 | 193 | 134 | 30 | 15 | 178/134/149 | Ferulic acid (*trans*) | Tent |  |  |
| Ferulic acid (*cis*) | 12.81 | 193 | 134 | 30 | 15 | 178/134/149 | Ferulic acid (*trans*) | Tent |  |  |
| Diferulic acid I | 14.51 | 385 | 341 | 40 | 15 | 341/326/282/297 | Ferulic acid (*trans*) | Tent |  |  |
| Diferulic acid II | 15.59 | 385 | 341 | 40 | 15 | 341/326/297/282/267 | Ferulic acid (*trans*) | Tent |  |  |
| Diferulic acid III | 17.86 | 385 | 341 | 40 | 15 | 341/326/282 | Ferulic acid (*trans*) | Tent |  |  |
| Diferulic acid IV | 18.77 | 385 | 341 | 40 | 15 | 341/193/178/149/134 | Ferulic acid (*trans*) | Tent |  |  |
| Diferulic acid (decarboxylated form) | 19.09 | 341 | 282 | 40 | 15 | 326/282/267/297 | Ferulic acid (*trans*) | Tent |  |  |
| Cinnamic acid | 19.14 | 147 | 103 | 45 | 15 | 103 | *p*-Coumaric acid *(trans*) | Tent |  |  |
| Triferulic acid I | 19.38 | 577 | 355 | 50 | 20 | 533/489/355/311/193 | Ferulic acid (*trans*) | Tent |  |  |
| Triferulic acid II | 19.75 | 577 | 355 | 50 | 20 | 355/193 | Ferulic acid (*trans*) | Tent |  |  |
| a SRM transition: single reaction monitoring used for quantification. b MS2 fragment: fragmentation products used for identification. c Std: Standard in which the phenolic has been quantified. When the phenolic was not quantified with its own standard, the quantification was tentative (Tent). LOD: limits of detection, LOQ: limits of quantification. | | | | | | | | | | |
